# Ophthalmic Education in Egypt

**Published:** 1919-09-06

**Authors:** 


### A MODERN CRUSADE.

### Ophthalmic Education in Egypt.

IN 1916 an article in this newspaper was devoted to the crusade against the ravages of ophthalmia which was being waged in Egypt. The campaign is of a permanent nature and the general staff, under the command of the Director of Ophthalmic Hospitals, Mr. A. F. MacCallan, F.R.C.S., depends on a process of attrition to wear down a scourge which is as old as the Pyramids. During the War, the forces fighting ophthalmia were cut down by the transfer of surgical personnel and equipment to Alexandria, to organise a temporary general hospital known as the Public Health Department Hospital at the suburb of Glymenopoulo; this was officially described by the Director of Medical Services as "the model of what a war hospital under canvas should be."

### A TYPICAL YEAR.

In the year 1918 the stable organisation of the hospitals permitted a more than pre-war standard of clinical material to be dealt with, when more than 90,000 new patients were examined, more than 900,000 attendances of patients were registered, and 54,000 operations were performed. This vast work was carried out at the 18 hospitals which have been established since in 1903 Sir Ernest Cassel made his gift of £40,000, which was the inception of the undertaking. Thirteen of the hospitals are specially designed and constructed for the sole purpose of ophthalmic surgery, the remaining five hospitals being travelling ophthalmic camps under canvas. The thirteen hospitals have been built at a cost of £55,000, which has been mainly raised in the towns and villages of Egypt by public subscription among the Egyptians themselves; they are maintained by public funds. It is interesting to learn that, in spite of the recent troubles in Egypt, a sum of £10,000 has been raised for the construction of a hospital in the province of Qalinbia since the rising of last March. This is eloquent of the desire of the Egyptians to procure ophthalmic treatment in the districts and of their regard for the disposal of the funds and the management of the hospitals by the Director.

It was by the decision of Lord Cromer that the earlier hospitals were received under the wing of the Department of Public Health and strengthened thereby on becoming Government institutions.

### TREATMENT OF CHILDREN.

The importance of obtaining treatment for babies and children attacked by ophthalmia is beginning to be recognised by the people. More than 7 per cent. of all the patients treated were under the age of one year, and 39 per cent. were under the age of fifteen years. School ophthalmic clinics are carried on at the Government primary schools and form the subject of a separate section of the Annual Report for 1918.

The personnel of the hospitals, except the Director, is entirely Egyptian, and the keenness of the surgeons in charge of hospitals is stated to be praiseworthy. It was these same surgeons who earned for the hospital for wounded at Glymenopoulo the commendation of the Director of Medical Services previously referred to. These surgeons are drafted voluntarily to the ophthalmic staff after obtaining their diplomas at the Government medical school; they then undergo two post-graduate courses of lectures from the Director in succeeding years, while during the whole period of two years' novitiate, they are kept under the tutelage of the Ophthalmic Inspectors and are not allowed to engage in private practice. Their professional attainments are such that the Egyptian Society of Ophthalmology, which includes the European ophthalmic surgeons in the country, publishes an annual report and is affiliated to the Ophthalmological Society of Great Britain.

There is a special ophthalmic, pathological and bacteriological laboratory to which are sent for examination specimens of all tumours removed, and also every eye excised.

The total cost of the hospitals and ophthalmic campaign, including all expenses of administration, amounts to £32,000 annually.

### WORK OF LAST TEN YEARS.

It is generally admitted that the ophthalmic conditions of the townsfolk and peasantry have been considerably ameliorated during the last ten years; nevertheless, 14½ per cent. of all the patients seen in 1918 were found to be blind in one or both eyes, showing that the ophthalmic campaign in Egypt is likely to be a long one, and to require a continuation of the ability and pertinacity with which it has been guided during the last sixteen years.

The annual report of the Ophthalmic Section of the Department of Public Health (obtainable at the Government Press, Cairo, price 2s. 1½d.) is again, as we stated in 1916, "a document worthy of careful study by economists, philanthropists, and statesmen, as well as by those interested in ophthalmology and in tropical hygiene. It contains much valuable information about the bacteriology of Egyptian ophthalmia, much of which is to all intents research work."

### AT HOME.

The news of this valuable ophthalmic work, together with the success which it has already met, comes most opportunely at a time when one reads of many pleas and resolutions for complete and perfect ophthalmic teaching in our home hospitals. It serves as an excellent illustration of the possible good which can come of thorough and extensive development of a so-called special department. The scant attention paid to certain of these subsidiary subjects has been notorious, and if our medical schools' past failings in this direction are not already universally obvious to teachers and students alike, then we would call attention to the work of the Egyptian ophthalmic hospitals.

